# How the Thermomechanical Processing Can Modify the High Strain Rate Mechanical Response of a Microalloyed Steel

**DOI:** 10.3390/ma14206062

**Published:** 2021-10-14

**Authors:** Remigiusz Błoniarz, Janusz Majta, Bogdan Rutkowski, Grzegorz Korpała, Ulrich Prahl, Jacek Janiszewski, Paulina Lisiecka-Graca

**Affiliations:** 1 Faculty of Metals Engineering and Industrial Computer Science, AGH University of Science and Technology, 30-059 Krakow, Poland; rutkowsk@agh.edu.pl (B.R.); graca@agh.edu.pl (P.L.-G.); 2 Institute of Metal Forming, TU Bergakademie Freiberg, 09599 Freiberg, Germany; grzegorz.korpala@imf.tu-freiberg.de (G.K.); ulrich.prahl@imf.tu-freiberg.de (U.P.); 3 Faculty of Mechatronics, Armament and Aerospace, Military University of Technology, 00-908 Warsaw, Poland; jacek.janiszewski@wat.edu.pl

**Keywords:** thermomechanical processing, microalloyed steel, dynamic loading, mechanical properties, microstructure development

## Abstract

The effects of thermomechanical processing (TMP) on the mechanical response of microalloyed steels subjected to dynamic loading conditions were examined. The deformation conditions in the thermomechanical laboratory rolling processes were selected on the basis of dilatometric tests. It allowed (with a constant value of total deformation) us to obtain microstructures with different compositions and morphology of the particular components. Several samples characterized by a particularly complex and unexpected representation of the obtained microstructures were selected for further research. Plastometric tests, i.e., compression and tensile tests, were performed under quasi-static loading with digital image correlation (DIC) analysis, and under dynamic loading on the Split Hopkinson Pressure Bar (SHPB) apparatus with strain rates of 1400 and 2000 s^−1^. Samples deformed in such conditions were subjected to microstructural analysis and hardness measurements. It has been observed that the use of various combinations of TMP parameters can result in the formation of specific microstructures, which in turn are the source of an attractive mechanical response under dynamic loading conditions. This opens up new possible areas of application for such popular structural materials which are microalloyed steels.

## 1. Introduction

The manufacturing of metal products in finished form or as semi-finished products with excellent quality and an optimal combination of mechanical properties has long been the main goal of technological development. Particularly high requirements exist for these products if they are intended to operate in extremely difficult conditions, e.g., where dynamic loads occur or may occur. Fortunately, the current state of knowledge allows for a properly selected chemical composition to design parameters of thermomechanical processing. This makes it possible to obtain the expected microstructures forming an attractive metallic composite of soft and hard components with the desired morphology. The current level of knowledge in the field of physical and mechanical metallurgy, and the continuous progress in the automation of metal forming processes, enable the use of precise settings of technological parameters and their control in industrial conditions.

A microalloyed steel, characterized by the increased strength due to the presence of Nb, V and Ti precipitates, is a well-known and widely used structural material [1,2]. Contrary to plain carbon steels, the microstructure development in these steel grades is very pronounced. Microalloyed steels are strengthened by precipitation, solid solution, work hardening, grain refinement mechanisms as well as multi-phase microstructure. The results of research published so far on the influence of thermomechanical processing conditions on microstructural effects of microalloyed steels show that it is possible to obtain a different mechanical response for the same chemical composition [3,4]. The most important ones, from the point of view of creating mechanical properties, are the composition of the microstructures and the morphology of individual components. It is commonly assumed that the most advantageous for obtaining good plasticity (toughness) and high strength (yield stress, YS; tensile strength, TS) is to guarantee a significant microstructure refinement. It turns out, however, that due to the appropriate phase composition, morphology of individual components and additional effects of controlled microstructural inhomogeneity, resulting from the specific arrangement of microstructural elements, it is possible to produce an attractive structural materials. Such materials are dedicated to withstanding specific loads, such as those observed under impact loading. It is already proved that reducing the average grain size and additionally the formation of the bimodal and complex phase (CP) microstructures lead to an optimization of the mechanical properties [3,4,5]. Further activity towards improving the mechanical properties, especially of microalloyed steels, may be the use of a bimodal microstructure in order to improve the ductility and plasticity of these materials under conditions of operation of very high strain rates [6,7,8,9]. One of the factors hindering the broad application of high-strength steel products is the apparent ductility decreasing, especially under dynamic loading conditions. This problem generally applies to body centered cubic crystal lattice (b.c.c.) metals whose strain rate sensitivity is high. Recently published works have suggested several ideas to improve the ductility of modern steels by using dispersed oxide, cementite or martensite [10,11,12], the use of a bimodal microstructure or distribution of grain size [13]. It is also possible to apply deformation of the ultrafine grained (UFG) material at a very low temperature and/or high strain rate, thereby increasing the high strain rate sensitivity or introducing heavy twinning into the material during processing [14]. Hence, it can be assumed that not only the microstructure refinement, but also the controlled and specific inhomogeneity of the complex microstructural components for a given chemical composition can be significant from the point of view of high strain rate mechanical response of the microalloyed steels. The main advantages of this approach are the avoidance of the need to use complex chemical compositions and the use of new technological processes, which is definitely more expensive compared to the production of already well-mastered processes for the manufacture of microalloyed high strength low alloy (HSLA) steels. There is still no substantial amount of research concerning the strain rate dependance of the mechanical response of the complex phase compositions of microalloyed steels. In particular, plasticity and strength at strain rate higher than 10^3^ s^−1^ (corresponding to dynamic loading condition) are important for applications in automotive bodies and in energy, naval or defense industries. The basic process parameters for obtaining a fine-grained and homogeneous microstructure of the microalloyed steels with particular chemical composition are soaking and deformation temperatures, prior austenite grain size, amount of deformation in the austenite non-recrystallization region and the cooling rates during and after the austenite transformation [15,16,17,18]. The recent progress and the latest achievements in thermomechanical processing of the first, second and third generations of advanced high-strength steels (AHSSs) and NANO (New Application of Nano Obstacles for dislocation movement) Nano Hiten steels have been very widely reviewed and thoroughly discussed in several published works, such as that of Zhao and Jiang [19].

The aim of the study was to investigate the influence of thermomechanical treatment on microstructural changes and the resulting mechanical response under high strain rate loading. Variations of all of the most important parameters of the thermomechanical processing of Nb-treated microalloyed steel were included in the applied laboratory rolling schemes. The evaluation of the influence of the obtained various microstructures for different histories of the deformation process was based mainly on the results of plastometric tests performed with the use of the Split Hopkinson Pressure Bar (SHPB) technique.

Summarizing, the main purpose of the present study is to clarify how the specific microstructures produced by the thermomechanical processing of Nb-microalloyed steel can affect strength and plasticity at very high strain rates. A wide and fully substantiated set of experimental tests of microalloyed steels is presented in this work. It may prove to be an indispensable tool for thermomechanical process control in order to produce new structural materials intended for applications under dynamic loading conditions.

## 2. Materials and Methods

**Figure 1:**
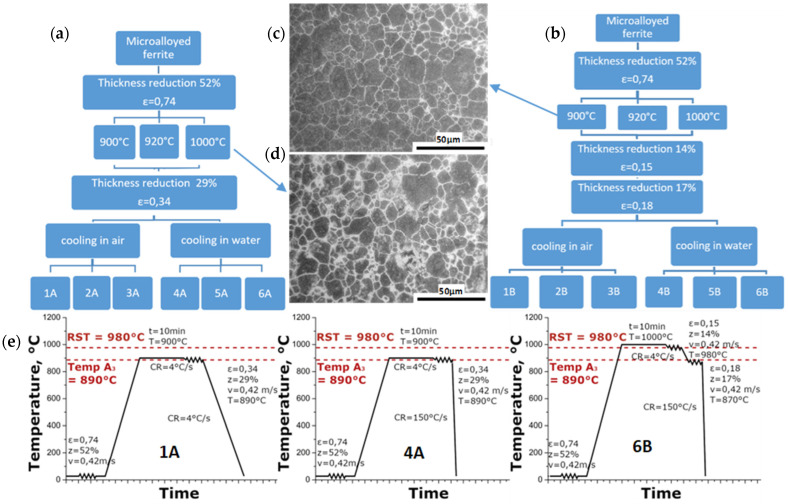
Schemes of applied thermomechanical treatment for schedule A (hot rolling with one pass) (**a**) and schedule B (hot rolling with two passes) (**b**). The initial microstructures of the austenite obtained after heating to 900 °C (**c**) and 1000 °C (**d**). Examples of thermomechanical rolling schemes for individual variants (**e**).

Microalloyed steels of 0.06 wt.% C, 0.058 wt.% Nb, 0.30 wt.% Si, 1.63 wt.% Mn, 0.045 wt.% V, 0.08 Mo, 0.02 wt.% Ti, 0.23 wt.% Ni and 0.006 wt.% N were examined. All the material specimens were made from commercial sheet with a thickness of 14.6 mm. The samples were first cold rolled on a 4-high laboratory rolling mill with a working roll diameter of ϕ 100 mm, at the Department of Metal Forming, AGH UST, Cracow, Poland. Total applied true strain was ε = 0.74 (thickness reduction z = 52% from 14.6 to 7 mm). Then, samples of ϕ 5 × 10 mm were cut according to longitudinal direction, and dilatometric tests were performed. Dilatometric tests as well as forming dilatometric tests were performed with the use of a quenching and forming dilatometer BÄHR Thermoanalyse GmbH (Hüllhorst, Germany) DIL 805 A/D equipped with an inductive heating system. The samples taken from cold-rolled flat bars were heated on a deformation dilatometer to the selected temperatures, 890, 900, 920 and 1000 °C, with a heating rate of 1 °C/s. The samples were held at the preset temperature for 3 min. Next, one sample for each temperature was subjected to compression with a strain equal to ε = 0.34, while the other sample was not deformed. After deformation, the samples were cooled to room temperature at the rate of 100 °C/s. On the basis of dilatometric tests, thermomechanical rolling diagrams (presented in Figure 1) were designed.

Microstructure studies were performed using Carl Zeiss AG (Oberkochen, Germany) Axio Imager M1m and Reichert Optical Company (Vienna, Austria) Metavar microscopes. Electron Back Scattering Diffraction (EBSD) analysis was performed using an FEI Company (Hillsboro, OR, USA) NanoSEM 450 microscope, with post-processing using Oxford Instruments (Witney, United Kingdom) Chanel 5 software. Each EBSD measurement was performed with a step size of 50 nm, and the limit for one grain qualification was 8 pixels. Additionally, in the grain size analysis, all grains with an area smaller than 0.2 µm^2^ were rejected for further processing. A Bruker Corporation (Billerica, MA, USA) AXS D8 Advance apparatus with a cobalt lamp generating X-rays of 1.7902 Å wavelength was used for X-ray diffraction (XRD) analysis.

Two groups of samples characterized by a particularly complex representation of the obtained microstructures were selected for further research. Under quasi-static conditions, the samples prepared from flat bars were examined under quasi-static tensile loading using Instron (Norwood, MA, USA) 4502, whereas the cylindrical specimens were used in compression tests using for tensile configuration, and the Zwick Roell Group (Ulm, Germany) Z250 machine was used for upsetting configuration. Tensile tests were accompanied by Dantec Dynamics (Skovlunde, Denmark) Q400 DIC strain analysis equipment. Hardness maps were made using Zwick Roell Group (Ulm, Germany) 3212 Hardness Tester.

**Figure 2:**
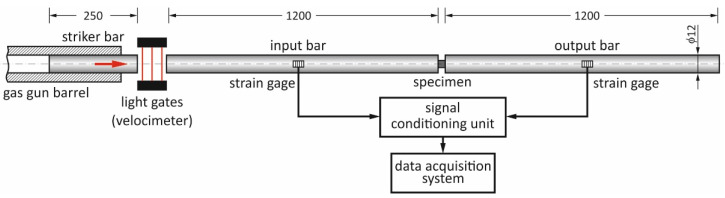
Scheme of the SHPB setup.

High strain rate compression tests were performed using the classical split Hopkinson pressure bar (SHPB) system built at the Military University of Technology, Warsaw, Poland. The basic parameters of the bar system shown in Figure 2 are as follows: input and output bar 1200 mm long, striker bar 250 mm long and all bars 12 mm in diameter. The bars were made of the heat treated maraging steel grade MS350 (yield strength—2300 MPa; Young modulus—196 GPa; elastic wave speed—4960 m/s). The striker bar was driven by a compressed air system with a barrel length and inner diameter of 1200 mm and 12.1 mm, respectively. The impact striker bar velocities applied during the experiments were between 9 and 21 m/s, ensuring strain rates between 150 and 2200 s^−1^ for the specified size of specimens used. Cylindrical samples of 4 mm length and 4 mm diameter were applied in all the SHPB experiments.

The pulse shaping technique was used to minimize the wave dispersion by damping the Pochhammer–Chree high-frequency oscillations and to facilitate stress equilibrium. The pulse shaper size was chosen for the given striker impact velocity and mechanical response of the tested material. It was found that for a given SHPB test condition, a copper pulse shaper with a 3 mm diameter and thickness ranging from 0.1 and 0.3 mm guarantees damping of the high-frequency oscillations and achieving dynamic stress equilibrium in the specimen. A grease based on MoS_2_ was applied to the contact interfaces between the specimen and the bars to minimize the interfacial friction.

The wave signals in the input and output bars were captured using strain gauges attached symmetrically to the opposite surfaces of the bars and in their middle length. The strain gauges were connected to a full Wheatstone bridge configuration. Typical electrical strain gauges of 1.6 mm gauge length were used (CEA-13-062UW-350, Vishay Micro Measurements Inc. [Raleigh, NC, USA]). The amplified signals of the strain gauges were recorded using a signal conditioning unit with frequency band of 1 MHz (SGA-0B V5 Wheatstone bridge with signal conditioning amplifiers, ESA Messtechnik, Olching, Germany) and a data acquisition system (LeCroy Chestnut Ridge [Chestnut Ridge, NY, USA], WJ354A high-speed digital oscilloscope).

Samples deformed during the plastometric tests were again subjected to microstructural analysis (optical microscopy, OM; transmission electron microscopy, TEM) and hardness measurements. Lamellae for the TEM observation were pre-pared with FEI Company (Hillsboro, OR, USA) SEM/Ga-FIB Helios NanoLab™ 600i. The TEM microscope used for structure analysis was FEI Company (Hillsboro, OR, USA) Tecnai G2 20 TWIN. Mapping of structural orientation based on diffraction patterns was performed by NanoMEGAS SPRL (Brussles, Belgium) ASTAR hardware with ACOM software.

## 3. Results and Discussion

Thermomechanical processing is a metallurgical process that integrates work hardening and heat treatment into a single process [20]. Thus, by using appropriate combinations of deformation history and cooling rates in the appropriate temperature ranges in austenite or during its phase transition, we can, for a given microalloyed steel with specified chemical composition, produce the expected mechanical properties that are primarily a function of the steel′s microstructure [21,22]. Generally, all microstructures obtained in the present study consist of ferrite/bainite matrix with a small amount of retained austenite, martensite and perlite. Nevertheless, the basic differences between the obtained materials result from the content of individual structural components and their specific morphology. On the other hand, favorable combinations of mechanical properties, including, in particular, strength and ductility under dynamic loading conditions, result from the structural inhomogeneity being a direct result of the thermomechanical processes carried out.

### 3.1. Dilatometric Tests

The main goal of the present study was to evaluate mechanical properties of samples with various, specifically prepared during the thermomechanical processing microstructures, deformed with high strain-rates. In order to precisely determine the critical temperatures for the austenitic range, the experiments began with dilatometric tests. Information was obtained on the starting temperature of the austenite–ferrite transformation for different reheating rates of the samples to the temperatures at which the rolling processes were carried out. The results obtained from the conducted tests are presented in Table 1. The key heating transformation temperatures were established by dilatometric testing at different reheating rates: 1, 10, 30, 50, 70 and 100 K/s. The approximate temperature of the end of the austenitic transformation of A_c1_ was taken as 880 °C. The differences in the duration of phase transitions resulting from the reheating rate turned out to be negligible from the technological point of view.

The austenite grain size is essential for the production of a specific final microstructure, strength and toughness of microalloyed steels after thermomechanical processing. Qualitatively and quantitatively, the austenite microstructure is already formed at the stage of reheating to the austenitizing temperature. Therefore, information from the dilatometric tests, which allowed for an assessment of the role of reheating rate, were investigated at the beginning of the present study.

**Figure 3:**
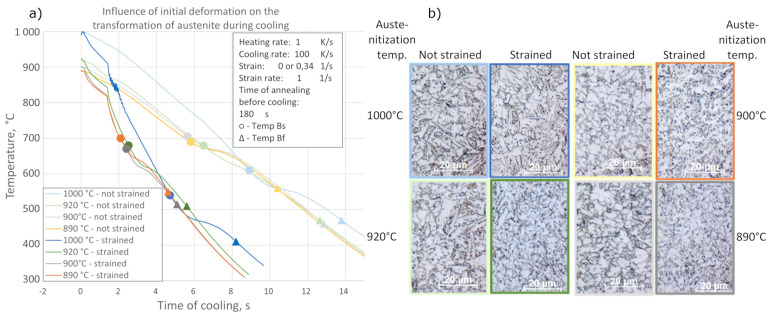
Influence of initial deformation on the course of dilatometric curves (**a**) and microstructures of the specimens (**b**).

Subsequent samples were tested in order to assess the influence of austenite deformation and austenitization temperature on phase transformations during cooling. Four austenitization temperatures were selected. After annealing (reheating rate 1 K/s) and austenitization (withstanding for 180 s), one sample was cooled and the other was deformed with a strain of 0.34, and then cooled. The cooling medium was nitrogen gas. Recorded dilatometric curves with assigned critical temperatures are presented in Figure 3, where the final microstructures after the tests are also presented.

A temperature of 890 °C has been chosen as the nearest temperature at which stable austenite exist. However, dilatometric tests do not correspond with real rolling processes because of the temperature drop during the transfer of the billet from the furnace to the rolls. This temperature drop is estimated as 10 °C. For this reason, an austenitization temperature of 900 °C was applied in order to achieve an 890 °C billet temperature during deformation.

During the laboratory rolling process, the billet size is relatively small compared to industrial processes, so the correct choice of temperatures may be disturbed due to the temperature fluctuations and non-uniform distribution that occur. Moreover, in order to assess the impact of the intensity of these changes on the kinetics of austenite transformation and the morphology of its products, an austenitization temperature of 920 °C was included in the adopted thermomechanical processing schemes. The influence of the austenite deformations at temperatures higher than austenite recrystallization (estimated as 980 °C, based on chemical composition) require higher austenitization temperature, so 1000 °C was chosen as the last test temperature. Two different kinds of cooling strategies were applied: in air and in water.

In the first stage of the conducted research, the images of microstructures and the resulting mechanical properties of samples deformed under different conditions of thermomechanical processing with the same total deformation were analyzed. Thus, for the same thickness of rolled flat bars, several structural materials of different properties were obtained. The thermomechanical treatment schemes used in the present study are presented in Figure 1. However, for the following investigations, we decided to study, more particularly, only selected schemes. On the basis of dilatometric tests, samples for thermomechanical rolling were prepared. Samples 1A, 1B, 3A, 3B, 4A, 4B, 6A and 6B were selected (rolling schedules—see Figure 1). The criterion for selecting samples for further tests resulted from the degree of differentiation of microstructures at room temperature and the process parameters used in the completed thermomechanical rolling tests.

### 3.2. Microstructural Studies

**Figure 4:**
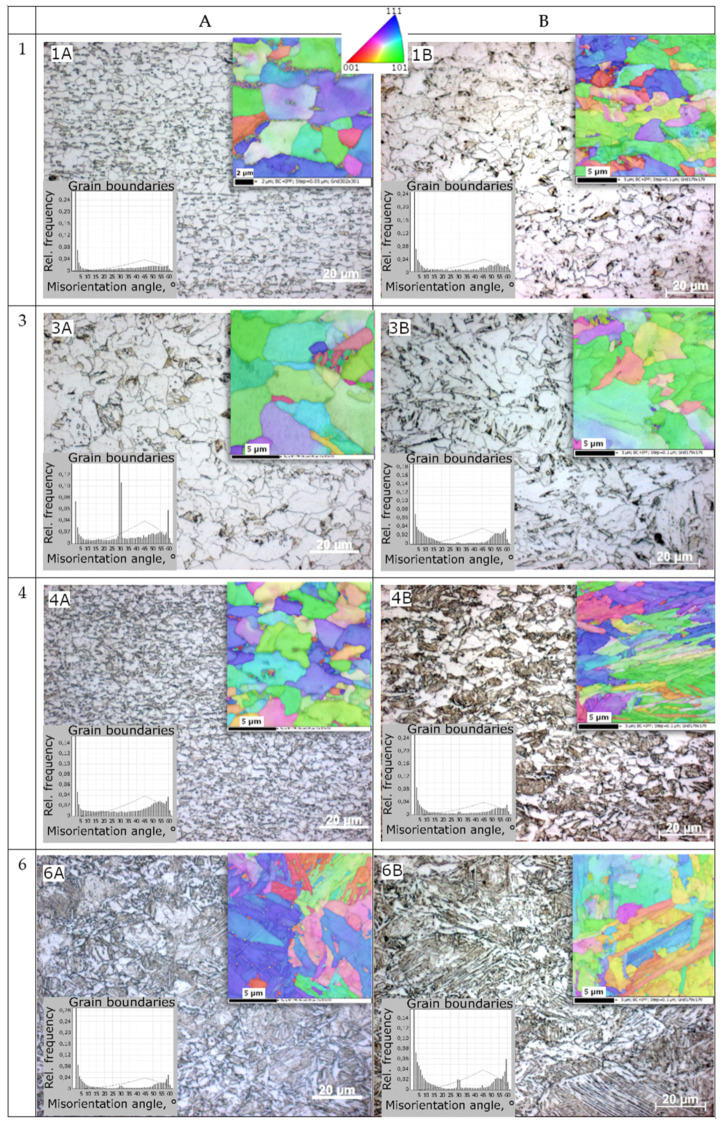
Microstructures of samples produced by various thermomechanical rolling. Microstructures obtained by optical microscopy and SEM using the EBSD technique.

Microstructural investigations of the specimens produced by the TMP were performed. Both the phase composition and the morphology of individual components of the studied microstructures were assessed. Examples of the results of the obtained images of microstructures are shown in Figure 4. The microstructural analysis was performed using samples cut, in a plane parallel to the rolling direction, from (i) the near surface (1 mm slice for prior austenite grain revealing due to low ability of that steel for martensitic transformation) and (ii) mid-thickness location of a 5 mm thick final flat bar. The microstructural characterization was carried out by optical and scanning electron microscopy (SEM) with EBSD technique. High mechanical properties, including impact toughness, require not only higher strength of the microstructure components, such as non-polygonal ferrite, acicular ferrite, bainites or martensite achieved due to dedicated chemical composition and high cooling rates, but also a controlled degree of their refinement and finally, in the case of many phases, of their proper arrangement in the micro-regions. Microstructural studies have revealed that bainite is the dominant microstructural component for the produced materials. Nevertheless, from the point of view of mechanical properties, an important factor characterizing the obtained microstructures is their heterogeneity and the morphology of individual components. It can be noticed (Figure 4) that in the case of samples 1A and 1B, the obtained microstructure is characterized by the highest proportion of ferrite with a fairly uniform degree of refinement, especially in the case of sample 1A. The greatest microstructural inhomogeneity can be seen in the images of samples 4A, especially 4B (Figure 4). The latter is dominated by a characteristic image of areas marked with granular ferrite, corresponding to the primary austenite structure, filled with a mixture of bainite, martensite and acicular ferrite. The microstructure revealed in sample 3A (Figure 4) shows a distinct bimodal character, i.e., there are uniformly distributed grains of very fine and much larger diameters. Such a microstructure, as already mentioned in [6,7,8,9], is very advantageous from the point of view of the optimal combination of ductility and high strength.

It should be expected that the observed characteristics of the microstructures, i.e., significant differences in morphology, phase composition and structure refinement, will have a significant impact on the mechanical responses of the tested samples under both quasi-static and dynamic loading conditions.

The quantitative assessment of the retained austenite was also the result of the per-formed analysis. The thermomechanically rolled specimens were examined using X-ray diffraction analysis (XRD). Based on the diffractograms, the volume fraction of retained austenite in the tested samples was calculated. Based on the evaluation of the microstructures, the volume fraction and the morphology of individual structural components and histograms showing the degree of disorientation within the grain boundaries, the dominant phases occurring in each of the microstructures were identified. The grain sizes presented in Table 2 were determined on the basis of maps from the EBSD analysis, where 0.2 µm^2^ was assumed as the minimum grain area.

### 3.3. Plastometric Tests

**Figure 5:**
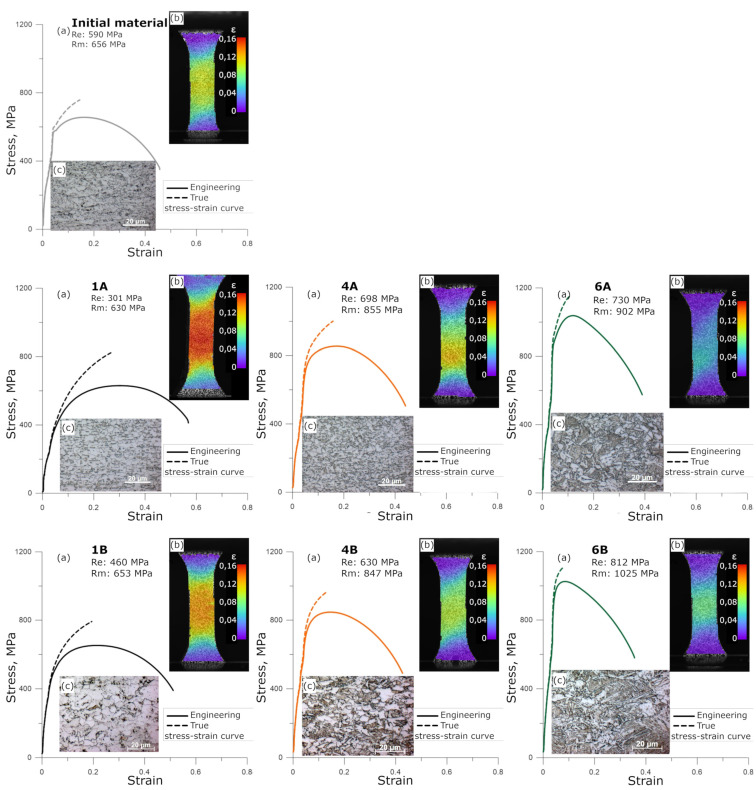
Examples of the quasi-static tensile test results (**a**). Measurement results and deformation distributions made by means of DIC (**b**). The representations of the initial microstructures (**c**) in the specimens are also presented.

On the basis of the microstructural tests performed and the aforementioned criterion of the selection of the research material, samples were selected for plastometric tests. In the first stage of the study, the quasi-static tensile tests were carried out. In addition to the obtained stress–strain curves, images of strain distributions were also produced using the DIC system. These tests allowed us to determine the mechanical characteristics of all tested samples under quasi-static loads. The examples of results of the tensile tests and measurements with the DIC system are shown in Figure 5. The representation of the initial microstructures in the samples before test is also shown.

As it was mentioned earlier, the microstructures obtained after thermomechanical rolling were formed mainly by ferrite–pearlite (specimens 1A, 1B) or bainite–martensite and acicular ferrite reaching (specimens 4A, 4B). The results presented in Figure 4 clearly show that hard microstructural components (perlite, bainite, martensite) exerted a significant influence on the mechanical response of the tested samples. These observations are consistent with literature [23,24] where it was shown that the ferrite was the soft microstructure component with high elongation, whereas bainite was the hard microstructure component with high strength. We can expect that in the high strain rate regime, the retained austenite (specimens 1A, 1B) can be considered as independent of strain rate in comparison to the ferrite and bainite, which usually show positive strain rate sensitivity.

**Figure 6:**
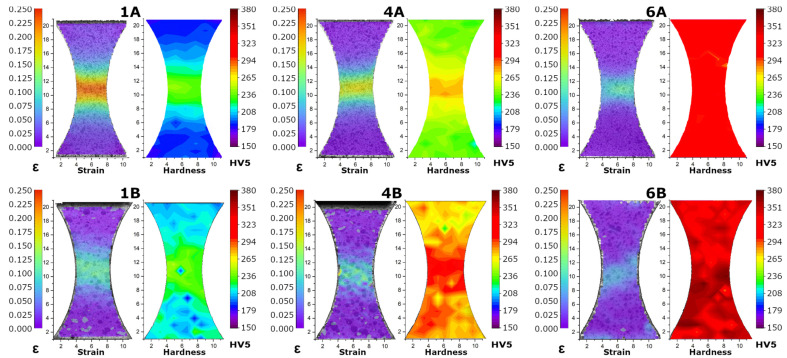
Maps of strain distributions and hardness in samples produced as a result of thermomechanical rolling. Specimens with a special, arcuate shape of the deformation zone were elongated until the maximum tensile force was reached.

An important feature of structural materials, especially those intended for applications in various load conditions due to the mechanical state, is their susceptibility to work hardening. For this reason, also in the case of the materials produced in this study after thermomechanical rolling, tests were carried out that allowed us to compare the mechanical response of individual samples in the function of deformation distribution and as a result of work hardening. First, the samples with a special shape (Figure 6) were subjected to the tension tests with this same range of the total elongations. During loading, the measurements of strain distributions were made using the DIC system [25], to determine the degree of the localizations of deformation. Then, the deformed samples were subjected to hardness measurements in order to assess the characterization of work hardening of the various microstructures. The results of the performed quasi-static tensile tests indicate significant differences in the ability of work hardening between the tested samples. Comparing samples 1A and 1B with 4A and 4B, it can be seen that in the case of the former, there is a stronger tendency for localization of the deformation. In the case of sample 6, in general, higher hardness values and weaker tendency for strain localization were observed.

It should be mentioned here that, as it is generally known, the formation of a local stress state at the micro level on various types of stress concentrators, e.g., hard phase and soft ferrite interfaces, facilitates the dissipation of deformation energy on a macro scale. The possibility of evaluating the characteristics of work hardening, which is presented in Figure 5 as an effect in the form of localization of both deformation and strengthening (hardness), is of key importance in this case. Therefore, it is reasonable to follow the relationships between the local inhomogeneous states of the microstructure, hard and soft components, and the resulting macro-scale consequences of the mechanical response. This dependence is of particular importance in dynamic load conditions, where the wave pattern of stress spreading is observed.

**Figure 7:**
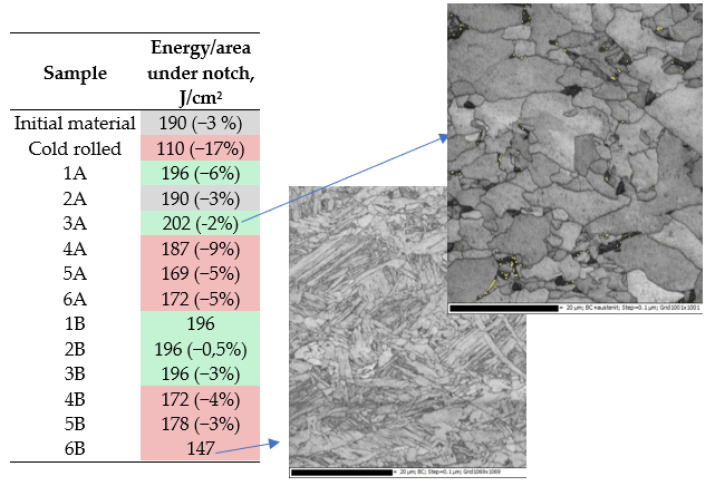
Energy consumed for breaking in Charpy test (impact toughness) for V-notched samples with not-normalized dimensions: width/thickness/length = 10/4/55 mm. The highest values of energy and percentage deviation in the negative are presented.

The next stage of the research included the measurements of impact toughness and the interpretation of the obtained results. It can be noticed (Figure 7) that in relation to the material in the initial state, based on the analysis of the fracture energy, the produced materials show a clear, dominant division in terms of cooling conditions. The samples cooled after rolling in air have a fracture energy slightly higher than or equal to that of the sample in the input state, while for samples cooled in water, this energy is much lower.

When analyzing the obtained results in more detail, it can be seen that in the case of thermomechanical rolling according to scheme A, samples 1A and 2A almost do not differ in the breaking energy from the input material. The increase in impact toughness was observed in the case of the sample rolled according to the scheme 3A and it should be noted that this increase is the greatest among all tested samples. Observing the image of the microstructure for this sample 3A, it should be assumed that high toughness value in this case is a direct result of the bimodal distribution of significantly refined and large grains [6,7,8,9,13]. The rolling pattern B (two hot passes), in turn, in each case shows an increase in the fracture energy for air-cooled samples, and the obtained results are very similar to each other. Thermomechanically rolled samples according to schemes 1B and 2B are characterized by high impact toughness, and this can be related to the morphology of their structure. The observed SEM fractures and the microstructures indicate a relatively uniform distribution of both larger and finely comminuted grains, which on the one hand ensures an increase in strength resulting from the refinement of the structure, and at the same time does not generate privileged places (as in the case of very fine grain conglomerates) for initiation and crack propagation.

**Figure 8:**
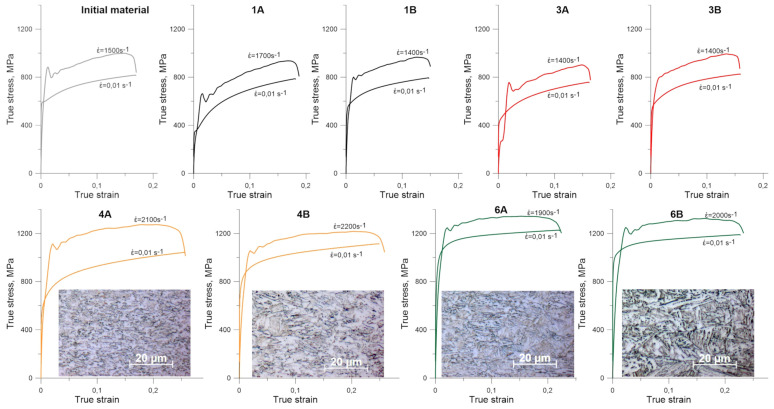
Quasi-static and high strain rate curves for microalloyed steel subjected to thermomechanical rolling. Microstructure images for water-cooled thermomechanical variants after high strain rate compression were added.

The influence of the strain rate on the mechanical properties of the tested samples was presented in Figure 8, in which the quasi-static and dynamic stress–strain curves obtained for samples subjected to different histories of thermomechanical treatment were collected.

Comparisons of the obtained results for samples subjected to different histories of thermomechanical treatment and subsequent compression in quasi-static conditions and SHPB are shown in Figure 8. The representation of the final microstructures in dynamically deformed samples is also shown. The microstructure analysis performed with the use of optical microscopy showed a uniform image of the microstructure on the sample cross-sections after dynamic deformation. This is consistent with the expectation that in the SHPB tests, the distribution of the effective strain on the sample cross-section should be as uniform as possible [26,27,28]. Using the comparisons of the obtained stress–strain curves for different strain rates, the increments of flow stress of the tested samples were determined as an effect of their strain rate sensitivity (Table 3). This seems to be the result of the finest refinement of the microstructure obtained with scheme 4A.

The selected stress–strain curves clearly show the possibility of obtaining significant changes in the mechanical properties of the tested microalloyed steels subjected to thermomechanical processing. It results directly from the fact that the HSLA steels are characterized by the highest number of strengthening mechanisms, i.e., deformation, solid solution, precipitation, complex phase and grain refinement strengthening. In turn, these strengthening mechanisms are activated in different ways in different microstructural compositions produced by a thermomechanical processing. Multiple microalloying additions also have a clear synergistic effect on hardenability. A favorable combination of high strength and ductility at high strain rates was obtained due to the developed multi-phase microstructure and its dedicated inhomogeneity in the micro and meso scales.

The results of plastometric tests presented in Figure 8 allow for the formulation of some general conclusions. The differences in the behavior of materials heated to 900 °C and 1000 °C (Figure 1) can be explained by the presence of more precipitates in the steel heated to a lower temperature, because the vanadium micro-additions did not dissolve sufficiently when reheated to the rolling temperature at 900 °C. In steel reheated to 1000 °C, the vanadium present in the solution did not have enough time to form carbides and the austenite with dissolved vanadium underwent a bainite transformation.

Sample 4A is characterized by the highest work hardening among water-cooled specimens. It contains the smallest share in the structure of upper bainite, and due to the lowest reheating temperature, it contains the highest number of carbides in the structure. At the given reheating temperatures, samples rolled in two passes (Figure 1) are characterized by higher strength in the quasi-static compressions than those rolled in one pass when cooled in the air. When cooled in water, single pass rolled specimens represent higher strength. The exception is sample 4A, for which the yield stress is lower than for sample 4B.

In the case of air-cooled samples, for high strain rate regime, the differences between samples rolled according to schemes A and B are the same as for quasi-static compression.

This is not the case with water-cooled samples. The increase in plastic flow stress of samples reheated before rolling to 1000 °C is the same, regardless of whether the rolling was carried out in one or two passes. In samples reheated before rolling to 900 °C, the increase in the yield stress of a sample rolled in one pass is much greater than in a sample rolled in two passes. The compression yield stress of the sample 4A at a strain rate of 2100 s^−1^ is higher than that of the sample 4B compressed at a strain rate of 2200 s^−1^.

Significant differences in the strain rate sensitivity were observed in case of samples 1A, 1B and 4A, 4B. The compression curves shown in Figure 8 also show that the sample 4A, characterized by a fine-grained structure of granular bainite strengthened with particles of foreign phases, shows the highest sensitivity to the strain rate while maintaining a high strain hardening ability. Hence, it can be concluded that for given strain rate and strain level, the microalloyed steel with heterogeneous, multiphase microstructure exhibits higher strain rate sensitivity than fully ferritic/pearlitic microstructure.

The analysis of the obtained results of compression tests clearly shows that material of sample 1A represents the highest work hardening rate, which may be due to the low yield stress associated with the presence of a relatively large amount of retained austenite. Additionally, the presence of a substantially high volume of retained austenite makes the sample material highly susceptible to work hardening.

Summarizing the results of compression tests presented in Figure 8, it should be stated that samples 1A, 1B and 4A, 4B exhibit an exceptionally attractive combination of mechanical properties under dynamic loading conditions. Sample 4A shows a higher sensitivity to strain rate even though the structure morphology is relatively similar to 1A, although sample 4A has a finer microstructure and sample 1A contains a significant amount of retained austenite. It follows that under dynamic load conditions, the grain refinement has a stronger positive effect on toughness than increasing the amount of retained austenite.

**Figure 9:**
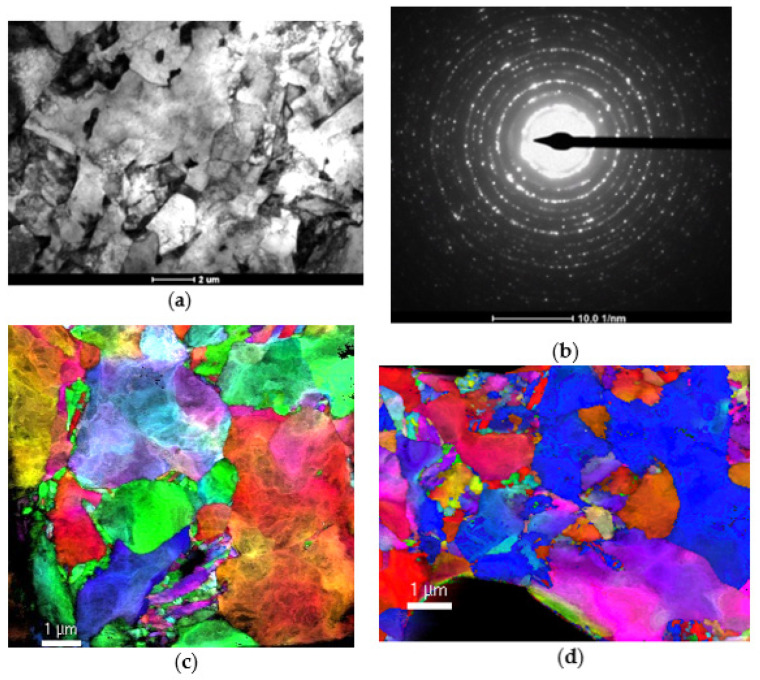
Scheme 4A after thermomechanical rolling: the TEM image (**a**) diffraction pattern (**b**) and maps of structural orientation after compression under quasi-static (**c**) and dynamic (SHPB –2000 s^−1^) (**d**) conditions.

At the end of the study, a more detailed analysis of the relationship between the history of thermomechanical rolling, the resulting microstructure and the mechanical properties, especially at high strain rates, was carried out. TEM microscopy and diffraction analysis were used in these studies. Sample images of the microstructures are shown in Figure 9.

Due to the very large differentiation of microstructural effects, selected cases were analyzed in detail. The microstructure analysis was carried out on samples made of rolled steel according to variant 4A (cold deformation, heating to 900 °C, rolling in one pass, cooling in water). The samples were analyzed at the condition after rolling and after deformation with the value of ε = 0.25 with two strain rates: 0.01 s^−1^ and 2100 s^−1^. TEM analysis showed that the original structure of sample 4A was composed of finely divided components with no visible signs of deformation. The diffraction pattern shows over 15 rings, formed out of numerous spots, the evidence of the large number of small grains. Such a complex diffraction pattern corresponds to a more complex microstructural features, than ferrite structure only. These rings are probably derived from numerous carbides. The dots on the diffraction pattern are clear and not stretched, which shows that the accumulated strain energy is not high and the material is relatively free from microstructural strain effects. The structure also includes bainite grains and numerous precipitates of various sizes, from a few to several hundred nm. The sample after deformation with a strain rate of 0.01 s^−1^ shows a strong development of the dislocation substructure and a large change in the crystallographic orientation within one grain. The presence of the precipitates facilitates the accumulation of microstructural effects of deformation. The dislocation substructure tends to be orderly. It is visible on the diffraction map, where the outlines of the subgrains can be seen. Dislocations are arranged in tangles, which transform into subgrain boundaries with further deformation.

The deformation effects can be observed much more significantly in the sample deformed with the strain rate of 2000 s^−1^. Optical microscope images of microstructures show that the microstructure is clearly finer than in the sample deformed at a lower strain rate. The change in the crystallographic orientation within the grains is smooth and does not form a distinct substructure. The photos of the dislocation substructure show the significant accumulation of dislocations blocked on numerous highly dispersive precipitates. This effect is characteristic for steels with Nb, Ti and V microalloying additions, and also for the microalloyed steel tested in this study.

The presented research has shown that it is possible to form the microstructure in the finished product in such a way as to achieve the expected mechanical responses at high strain rates without the need to change the chemical composition of the microalloyed steel. For this reason, further study on the problem discussed in this paper should be directed towards the creation of appropriate models that can be used directly for designing an online control of more and more complex thermomechanical processing of the microalloyed steels. The results of the present study are important not only for the automotive, defense and mining industries, but also for the further development of processes under dynamic forming conditions.

## 4. Conclusions

In the present study, a series of thermomechanical rolling tests and plastometric tests at low and high strain rates were presented. The main conclusions may be drawn based on the investigations of 0.06 wt.% C, 0.058 wt.% Nb, 0.30 wt.% Si, 1.63 wt.% Mn, 0.045 wt.% V, 0.08 Mo, 0.02 wt.% V, Ti, 0.23 wt.% Ni and 0.006 wt.% N microalloyed steel. These compositions were selected to provide representation of the commonly used grade X70. The basic feature of the tested steel is the presence of two main strengthening mechanisms, i.e., precipitation strengthening due to Nb, V and Ti, as well as grain refinement. Multiple microalloying additions also have a clear synergistic effect on hardenability. A favorable combination of high strength and ductility at high strain rates was obtained thanks to the developed multi-phase microstructure and its dedicated inhomogeneity in the micro and meso scales. The paper presents several possible strategies for modifying the dynamic response of the examined microalloyed steels. Depending on the boundary conditions of the thermomechanical process, it is possible to change the history of the processing, i.e., reheating temperature, deformation schedule, cooling rate, or to change the finishing temperature from conventional austenite rolling to austenite–ferrite intercritical rolling, or a combination of both. The presented research results and their discussion clearly indicate the need for further research for selected histories of thermomechanical processing in order to more precisely determine the changes in the strain rate sensitivity of the studied microstructures. The obtained results, both from the study of mechanical responses and microstructural analyzes, allow for the formulation of the following conclusions.

1. The microstructural and plastometric tests performed showed that under dynamic loading conditions, the refinement of the grains has a stronger positive effect on toughness than the increase in the amount of retained austenite.
2. It has been shown that the bimodal and gradient microstructures with large fraction of bainite/martensite clusters surrounded by ultra-fine-grained ferrite are capable of producing steels with an attractive combination of strength and ductility at high strain rates.
3. Observing the image of the microstructure for the sample 3A, it should be assumed that good toughness in this case is a direct result of the bimodal distribution of significantly refined and large grains.
4. The analysis of the obtained results of plastometric tests clearly shows that sample 1A represents the highest work hardening rate, which may be due to the low yield stress associated with the presence of a relatively large amount of retained austenite. Additionally, the presence of a substantially high volume of retained austenite makes the sample highly susceptible to work hardening.
5. For given strain rate and strain, the microalloyed steel with heterogeneous, multiphase microstructure exhibits higher strain rate sensitivity than fully ferritic/pearlitic microstructure. This is a suggestion for the further development of new high-strength ultra-fine-grain steels that can exhibit attractive mechanical behavior under dynamic loading.

## Figures and Tables

**Table 1:** Critical temperatures determined during reheating at different rates.

	Transformation during Reheating up to 1200 °C					
	1 K/s	10 K/s	30 K/s	50 K/s	70 K/s	100 K/s
A_c1_, °C	800	820	820	820	820	825
A_c3_, °C	892	870	880	880	883	885

**Table 2:** The results of the analysis of microstructure components for the applied thermomechanical rolling schemes (Figure 1) and the quantitative assessment of the degree of refinement as well as the amount of retained austenite.

Scheme	Microstructure Components	Average Grain Diameter (EBSD), µm	Retained Austenite Fraction, %
1A	Granular bainite	2.28	5.73
1B	Granular bainite	2.33	3.47
2A	Granular bainite	2.23	2.83
2B	Granular bainite/upper bainite	2.12	4.2
3A	Granular bainite	2.19	1.12
3B	Upper bainite	2	4.88
4A	Granular bainite	1.76 (1.67 µm taken from thin film from TEM)	
4B	Upper bainite	1.99	
5A	Upper bainite	1.89	
5B	Upper bainite	2.02	
6A	Upper bainite/lower bainite	1.73	
6B	Upper bainite/lower bainite	1.71	

**Table 3:** Increases in the flow stresses due to the strain rate sensitivities at strain 0.1 for stress–strain curves presented in Figure 8.

Sample	Flow Stress under Quasi-Static Loading	Flow Stress under Dynamic Loading	Increase in the Flow Stress	Strain Rate Sensitivity, m
6A	1190	1332	142	0.0093
6B	1150	1300	150	0.0100
4A	928	1229	301	0.0229
4B	1035	1177	142	0.0105
3A	703	841	138	0.0151
3B	786	955	169	0.0164
1A	698	849	151	0.0163
1B	759	935	176	0.0176
Initial material	770	961	191	0.0186

## Data Availability

Not applicable.

## References

[1] Villalobos J.C., Del-Pozo A., Campillo B., Mayén J., Serna S. (2018). Microalloyed Steels through History until 2018: Review of Chemical Composition, Processing and Hydrogen Service. Metals.

[2] DeArdo A.J. (2003). Niobium in modern steels. Int. Mater. Rev..

[3] Muszka K., Hodgson P., Majta J. (2006). A physical based modeling approach for the dynamic behavior of ultrafine grained structures. J. Mater. Process. Technol..

[4] Ardehali Barani A., Li F., Romano P., Ponge D., Raabe D. (2007). Design of hogh-strength steels by microalloying and thermomechanical treatment. Mater. Sci. Eng. A.

[5] Song R., Ponge D., Raabe D., Speer J., Matlock D. (2006). Overview of processing, microstructure and mechanical properties of ultrafine grained bcc steels. Mater. Sci. Eng. A.

[6] Song R., Ponge D., Raabe D. (2005). Mechanical properties of an ultrafine grained C–Mn steel processed by warm deformation and annealing. Acta Mater..

[7] Majta J., Zurek A., Pietrzyk M. (1997). The Effect of Deformation in the Two-Phase Region of C-Mn and Microalloyed Steels on the Mechanical Behavior of the Resulting Structure. J. Phys. IV Fr..

[8] Storojeva L., Ponge D., Kaspar R., Raabe D. (2004). Development of microstructure and texture of medium carbon steel during heavy warm deformation. Acta Mater..

[9] Saito Y. (1987). Mathematical model of hot deformation resistance in austenite-ferrite two phase region. Trans. Iron Steel Inst. Jpn..

[10] Belyakov A., Sakai Y., Hara T., Kimura Y., Tsuzaki K. (2004). Effect of Nano-Sized Oxides on Annealing Behaviour of Ultrafine Grained Steels. Mater. Trans..

[11] Tsuji N., Ueji R., Minamino Y., Saito Y. (2002). A new and simple process to obtain nano-structured bulk low-carbon steel with superior mechanical property. Scr. Mater..

[12] Huyashi T., Nagai K. (2002). Improvement of strength-ductility balance for low carbon ultrafine-grained steels through strain hardening design. Trans. Jpn. Soc. Mech. Eng..

[13] Benito J.A., Tejedor R., Rodríguez-Baracaldo R., Cabrera J.M., Prado J.M. (2009). Ductility of Bulk Nanocrystalline and Ultrafine Grain Iron and Steel. Mater. Sci. Forum.

[14] Narayana Murty S.V.S., Torizuka S. (2010). Mechanical properties of ultrafine grained steels processed by large strain-high Z deformation—A review. Mater. Sci. Forum.

[15] Majta J., Lenard J.G., Pietrzyk M. (1996). Modelling the Evolution of the Microstructure of a Nb Steel. ISIJ Int..

[16] DeArdo A.J., Hua M.J., Cho K.G., Garcia C.I. (2009). On strength of microalloyed steels: An interpretive review. Mater. Sci. Technol..

[17] Senuma T. (2001). Advances in Physical Metallurgy and Processing of Steels. Physical Metallurgy of Modern High Strength Steel Sheets. ISIJ Int..

[18] Varlinden B., Driver J., Samajdar I., Doherty R.D. (2007). Thermo-Mechanical Processing of Metallic Materials.

[19] Zhao J., Jiang Z. (2018). Thermomechanical processing of advanced high strength steels. Prog. Mater. Sci..

[20] DeGarmo E.P., Black J.T., Kohser R.A. (2003). Materials and Process in Manufacturing.

[21] Prasad S., Sarma D. (2005). Influence of thermomechanical treatment on microstructure and mechanical properties of a microalloyed (Nb + V) weather-resistant steel. Mater. Sci. Eng. A.

[22] Chen J., Tang S., Liu Z.-Y., Wang G.-D. (2013). Microstructural characteristics with various cooling paths and the mechanism of embrittlement and toughening in low-carbon high performance bridge steel. Mater. Sci. Eng. A.

[23] Slycken V.J., Verleysen P., Degrieck J., Bouquerel J., De Cooman B.C. (2007). Dynamic response of aluminum containing TRIP steel and its constituent phases. Mater. Sci. Eng. A.

[24] Khan A.S., Baig M., Choi S.-H., Yang H.-S., Sun X. (2012). Quasi-static and dynamic responses of advanced high strength steels: Experiments and modeling. Int. J. Plast..

[25] Graca P., Muszka K., Majta J., Perzyński K. (2016). Digital Image Correlation (DIC) System as a Verification Tool for Constitutive Models of Deformation with Complex Strain Path Changes. Comput. Methods Mater. Sci..

[26] Gama B.A., Lopatnikov S.L., Gillespie J.W. (2004). Hopkinson bar experimental technique: A critical review. Appl. Mech. Rev..

[27] Pankow M., Attard C., Waas A.M. (2009). Specimen size and shape effect in split Hopkinson pressure bar testing. J. Strain Anal. Eng. Des..

[28] Pei P., Pei Z., Tang Z. (2020). Numerical and Theoretical Analysis of the Inertia Effects and Interfacial Friction in SHPB Test Systems. Materials.

